# Reliability and Accuracy of a Time-Efficient Method for the Assessment of Achilles Tendon Mechanical Properties by Ultrasonography

**DOI:** 10.3390/s22072549

**Published:** 2022-03-26

**Authors:** Steve Hunter, Julian Werth, Darren James, Yiannis Lambrianides, Kenton Smith, Kiros Karamanidis, Gaspar Epro

**Affiliations:** Sport and Exercise Science Research Centre, School of Applied Sciences, London South Bank University, London SE1 0AA, UK; werthj2@lsbu.ac.uk (J.W.); jamesd6@lsbu.ac.uk (D.J.); lambriy2@lsbu.ac.uk (Y.L.); smithk41@lsbu.ac.uk (K.S.); k.karamanidis@lsbu.ac.uk (K.K.); g.epro@lsbu.ac.uk (G.E.)

**Keywords:** tendon elongation, tendon strain, tendon stiffness, muscle strength

## Abstract

The assessment of the force–length relationship under mechanical loading is widely used to evaluate the mechanical properties of tendons and to gain information about their adaptation, function, and injury. This study aimed to provide a time-efficient ultrasound method for assessing Achilles tendon mechanical properties. On two days, eleven healthy young non-active adults performed eight maximal voluntary isometric ankle plantarflexion contractions on a dynamometer with simultaneous ultrasonographic recording. Maximal tendon elongation was assessed by digitizing ultrasound images at rest and at maximal tendon force. Achilles tendon stiffness index was calculated from the ratio of tendon force-to-strain. No within- and between-day differences were detected between the proposed method and manual frame by frame tracking in Achilles tendon maximal force, maximal elongation, maximal strain, and stiffness index. The overall coefficient of variation between trials ranged from 3.4% to 10.3% and average difference in tendon tracking between methods was less than 0.6% strain. Furthermore, an additional assessment demonstrated significant differences between elite athletes, healthy young, and older adults in Achilles tendon force and stiffness index. Hence, the analysis has the potential to reliably and accurately monitor changes in Achilles tendon mechanical properties due to aging and altered mechanical loading in a time-efficient manner.

## 1. Introduction

The assessment of the force–length relationship under mechanical loading is a common and acknowledged technique to determine the mechanical properties of biological tissues. This is particularly important for tendons as their mechanical characteristics are known to influence the force and power generation capacities of muscle-tendon units during functional activities. Further, the examination of tendon mechanical behavior during loading can provide relevant information with respect to changes in tendon biomechanical properties resulting from tendon injury, aging, and altered mechanical loading. Thus, well-established methods to assess in vivo human tendon mechanical behavior during loading are warranted to investigate tendon mechanical properties and function in physiological and pathological conditions. The analysis of human Achilles tendon (AT) mechanical properties in vivo using ultrasonography combined with dynamometry has established itself as a non-invasive, affordable, and easily applied valid measure. The application of the ultrasound method has provided relevant information with respect to tendon adaptation [1,2], tendon injury [3], and tendon function [4] across different populations and age groups. For example, repeated exposure to specific mechanical loading (e.g., resistance training) can increase tendon stiffness along with muscle strength gains [1,2], whereas tendinopathic as well as aging tendons demonstrate lower stiffness and higher tendon strains at given force levels [3,5,6]. Recent observations indicate that due to discrepancies between the time frames of muscle and tendon adaptation, imbalances between muscle force generation and tendon resistance may occur through periods of exercise, potentially increasing the demand of the tendon and its risk to overuse injuries [7]. Hence, being able to assess human muscle-tendon unit biomechanical properties in vivo could be of importance for clinical and sport settings.

AT elongation during muscular contraction is usually estimated by choosing a tissue landmark (e.g., myotendinous junction or muscle fascicle insertion) using ultrasonography and digitizing that landmark frame by frame [1,3,8]. Based on the generated force–length relationship, AT stiffness is commonly assessed by linear regression using intervals from 50% to 100% [8], 80% to 100% [9], or from 90% to 100% of the calculated AT force [10]. However, previous phantom experiments have indicated that the accuracy of tendon elongation estimated by ultrasonography is on average about ±1 mm [4,11]. When further considering that the gastrocnemius medialis (GM) myotendinous junction elongation in the higher force regions is rather low (about 2–4 mm) [12], it might lead to a considerably resultant high error (potentially even up to 50%). Although it is known that the in vivo AT force–length relationship determined by ultrasonography is not linear (due to the toe region), most studies have observed a similar shape before and after an intervention (e.g., resistance exercise [8]) and between different subject groups (e.g., older vs. younger [6]). Therefore, AT stiffness may alternatively be estimated based on the stiffness index, which is defined as the ratio of AT force to elongation [13,14]. Determining AT elongation from 0% to 100% of the calculated AT force also has the advantage of reducing the potential relative error in comparison to using only the higher regions of the force–elongation relationship. Moreover, if the maximal AT elongation could be reliably and accurately assessed via only two ultrasound frames (i.e., at rest and at maximal AT force) instead of digitizing a tissue landmark frame by frame, an immediate assessment of AT mechanical properties would be possible for clinical and sports settings; even with limited computational skills. Once examined for its reliability and accuracy, such an analysis would provide an alternative time-efficient assessment of human AT mechanical properties in vivo for application in clinical, sport, and scientific settings to monitor the time course of changes in AT mechanical properties resulting from injury, maturation, aging, and altered mechanical loading.

Therefore, the purpose of this study was to investigate the reliability, accuracy, and sensitivity of a time-efficient analytical method for assessing AT mechanical properties using ultrasonography in three experiments. In one experiment, we examined the within- and between-day reliability of AT mechanical properties by digitizing ultrasound frames at rest and at maximal AT force using a semi-automatic analysis software. In a second experiment, the method was indirectly validated against the manual tracking approach of digitizing ultrasound images frame by frame throughout the time-course of a plantarflexion contraction. A third experiment involved the assessment of AT mechanical properties in young non-active subjects, young elite athletes, and older individuals to examine whether the method is sensitive enough to determine differences in AT mechanical properties between populations.

## 2. Materials and Methods

### 2.1. Subjects

Eleven healthy young male non-active adults (means and SD; age: 28 ± 6 years; body height: 179 ± 4 cm; body mass: 75.5 ± 7.8 kg), eight young male elite athletes (24 ± 2 years; 191 ± 7 cm; 79.6 ± 5.2 kg), and eight non-active older male adults (68 ± 1 years; 176 ± 4 cm; 67.3 ± 3.1 kg) participated in the study. The elite athletes were at (inter)national level and recruited from sporting activities involving high AT strain magnitude (two pole vaulters, one long jumper, three high jumpers, and two sprinters). Most of the young non-active and older individuals were not involved in any regular sporting activity, except three young and two older subjects who were recreationally training once or twice a week. However, these exercise sessions involved low-magnitude AT mechanical stress or strain (e.g., swimming and yoga), which cannot be considered as an appropriate stimulus to cause adaptive changes in triceps surae muscle-tendon unit mechanical properties. The study was approved by the university’s ethics committee and informed consent was given by all subjects.

### 2.2. Experimental Setup

**Figure 1:**
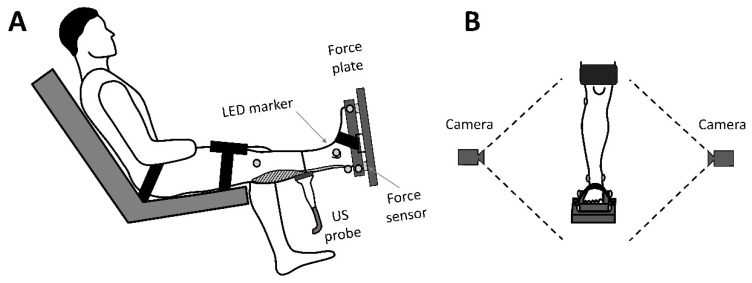
The used custom-built dynamometer and measurement setup: (**A**) The participant was positioned with the foot on the strain-gauge load cell-based dynamometer (force plate) with their shank perpendicular to the foot and knee fully extended. (**B**) Two cameras were used to track four active LED markers on the lower extremity (head of the fibula, malleolus lateralis, malleolus medialis, and calcaneus) and four markers fixed on the force plate footplate at predefined locations (two each side).

The experimental setup used in this study has been described in detail previously [15,16]. Briefly, after warming up (hopping and stretching for three minutes) the subjects were seated on a custom-built dynamometer with the shank perpendicular to the foot and the knee fully extended (neutral position; Figure 1). A custom-made fixation, built out of the material of a ski boot buckle-catch system, was applied around the foot and the dynamometer foot plate to reduce any joint motion during contraction. Ten submaximal and one maximal isometric voluntary ankle plantarflexion contractions were performed by each subject for familiarization and AT preconditioning [17]. Following warm-up, the participants were given extra resting time (3–4 min) to avoid any potential fatigue effects from the tendon preconditioning. For the reliability analysis, only the young non-active subjects were considered. These subjects had to perform four ramp contractions with the same leg on the dynamometer on two consecutive days (day 1 and day 2). For our subject-group comparison, the elite athletes and older adults performed four contractions with each leg on one day.

The reaction forces under the foot during contraction were determined by three strain gauge load cells fixed at predefined distances on the foot plate (100 Hz; Figure 1A). Eight light-emitting diodes (LEDs) were used as active markers to examine kinematics. Four active markers were placed on the lower extremity (head of the fibula, malleolus lateralis, malleolus medialis, and calcaneus) and four markers were fixed on the force plate at predefined locations. Two cameras (Basler, Germany, 15 Hz; Figure 1B) were used to record the markers in the sagittal plane to assess the inevitable ankle joint changes during the plantarflexion contractions. The 2D trajectories of the markers were automatically tracked frame by frame by the TEMULAB software (TEMULAB^®^, Protendon GmbH & Co. KG, Aachen, Germany) [15].

The elongation of the GM myotendinous junction during contraction was visually reproduced using a linear array ultrasound probe (Aloka α7, Tokyo, Japan) and stored on the computer at 30 Hz using a frame grabber (Epiphany, Ontario, CA, USA). The tendon’s resting length was determined for each subject and day at neutral position as the distance between the GM myotendinous junction and the tuberositas calcanei (both determined using US and marked with a thin black tape) along the skin surface by using a digital measuring wheel device (K&R, online5, Nuremberg, Germany). The black tape at the GM myotendinous junction was further used to check for any displacement of the probe relative to the skin during contractions. To synchronize the different signals, two LEDs, a transistor–transistor logic (TTL) signal and an optical trigger on the ultrasound videos were used. All trigger signals were automatically identified using the TEMULAB analysis software, allowing a real-time synchronization of the used measurement systems.

### 2.3. Analysis of AT Mechanical Properties

All captured ultrasound videos were analyzed using the TEMULAB software on two ultrasound frames: one at rest and one at maximal AT force. For the analysis, a landmark at the myotendinous junction of the GM was identified by the software at rest (before contraction) and at 100% of the calculated AT force; and where necessary, was manually corrected by the operator. Since the synchronization of all signals and the AT force calculation were performed in real-time, the ultrasound tracking procedure could be completed immediately after each measurement. To indirectly validate the method, another operator manually digitized the eighty-eight ultrasound videos from the eleven young non-active subjects (eight videos per subject) frame by frame using the Simi video analysis system (Simi Motion 6.1; Simi Reality Motion Systems GmbH, Unterschleißheim, Germany).

AT force was calculated as dividing the ankle joint moment by the AT moment arm. The AT moment arm was estimated for each individual by the perpendicular distance from the ankle joint’s center of rotation (i.e., axis through the inferior tip of the medial and lateral malleoli) to the AT according to the palpation method proposed by Scholz and colleagues [18]. This moment arm was further used to estimate the tendon elongation due to the inevitable ankle joint rotation during contraction [10,19] using the equation of tendon excursion method [20,21] (i.e., tendon excursion was obtained from the product of the moment arm and the changes in ankle joint angle). In this way, the actual tendon elongation due to the exerted tendon force could be estimated. AT strain was determined by dividing the AT elongation by the tendon resting length. For both methods (simplified two-frame method and frame-by-frame method) the normalized AT stiffness index was then assessed using the ratio of AT force-to-strain from 0% to 100% of AT force. The ratio between AT force and strain was used since differences in the tendon’s resting length between subject groups or between measurements would affect the estimation of AT stiffness (relationship between the calculated AT force and elongation).

### 2.4. Statistics

Kolmogorov–Smirnov tests confirmed for the Gaussian assumption (*p* > 0.05 after Lilliefors correction) for all examined parameters (AT maximal force, maximal elongation, maximal strain, and normalized stiffness index). To determine within- and between-day reliability of those parameters, the eight trials derived from the first (Trial 1–Trial 4) and second day (Trial 5–Trial 8) of the non-active young adult subject group were used. Firstly, a two-way mixed measures analysis of variance (ANOVA; with trial and day as factors) was used to determine any differences in the analyzed parameters. To determine the differences in absolute values within- and between-day, as well as overall (across all measurement trials), the root mean square (RMS) differences was used. The reliability between measures (within- and between-day and overall) was examined using the intraclass correlation coefficient (ICC; absolute agreement and single measures), with the values defined as “poor” (<0.50), “moderate” (0.50–0.75), “good” (0.75–0.90), and “excellent” reliability (>0.90) [22,23]. The least number of trials needed to provide reliable parameter values was estimated with the Spearman–Brown prophecy formula (acceptable level of confidence was set at 0.9). Further, the coefficient of variation (CV) was applied for each subject and parameter across trials to test the typical error expressed as a percentage of the subject’s mean score within-day and overall.

The accuracy of the proposed method in comparison to the frame-by-frame method in the measured maximal AT strain (i.e., without correcting for AT strain caused by the inevitable ankle joint rotation) was performed using a univariate ANOVA with method as a factor. To determine the difference in absolute size and to examine the strength of relationship in maximal AT strain between methods, RMS and the Pearson correlation coefficient, respectively, were used.

In order to assess the sensitivity of the proposed method, a comparison of AT properties between subject groups (older adults, young non-active adults, and elite athletes) was performed using a univariate ANOVA. If there were significant effects, a post hoc Bonferroni correction was performed to locate possible differences between pairs. In order to decrease the power of our comparison, irrespective of the leg, the average value of the four strongest contraction trials of the older adults (highest maximal AT force); the average value of the four weakest contraction trials of the elite athletes (lowest maximal AT force); and the average value of all eight trials of the same leg in young non-active subjects were considered. The level of significance was set at α = 0.05.

## 3. Results

### 3.1. Within- and Between-Day Reliability

On day one, on average a maximal AT force of 4351 ± 865 N (means ± SD) caused a maximal AT elongation of 13.1 ± 2.9 mm, and a maximal AT strain 6.3 ± 1.5% (Table 1). On day two, the corresponding values were 4521 ± 931 N, 12.4 ± 2.5 mm, and 6.0 ± 1.1%. Normalized AT stiffness index was 71.6 ± 21.0 kN · strain^−1^ and 76.2 ± 15.0 kN · strain^−1^ on day one and day two, respectively. There were no significant within- or between-day effects on the analyzed parameters (*p* > 0.05; Table 1) and CV ranged between 3.4% (AT force on day two) and 10.3% (overall maximal AT elongation; Table 2).

AT maximal force, maximal elongation, maximal strain, and stiffness index showed RMS differences of 344 N, 1.4 mm, 0.6%, and 8.7 kN · strain^−1^ within-day (average across the days), and 534 N, 2.3 mm, 1%, and 10.6 kN · strain^−1^ between-day (Table 3). ICC within- and between-day were 0.93 and 0.90 for maximal AT force, 0.87 and 0.56 for maximal AT elongation, 0.87 and 0.72 for maximal AT strain, and 0.86 and 0.75 for normalized AT stiffness index (Table 4). Further, it was found that two to three contraction trials were required to provide reliable AT mechanical characteristics, except for the maximal AT elongation (where five trials were required; Table 4).

### 3.2. Accuracy of the Proposed Method

**Figure 2:**
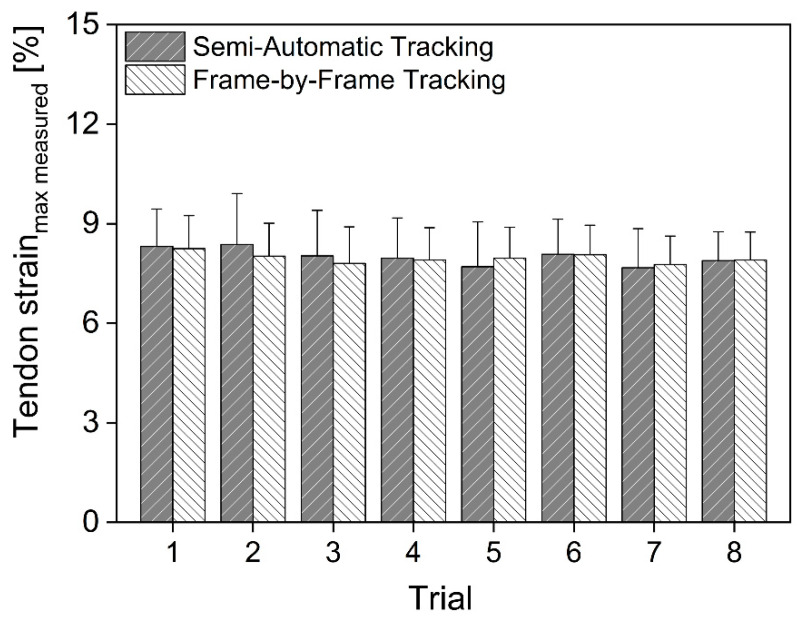
Means and SD of the measured maximal Achilles tendon strain (i.e., without correcting for tendon strain caused by the inevitable ankle joint rotation) for the devised method (i.e., digitizing US images at rest and at maximal AT force) and for the manual frame-by-frame tracking procedure for all eight trials.

The comparison of measured maximal Achilles tendon strain during contraction assessed by the semi-automatic analysis using two frames and by manually digitizing ultrasound images frame by frame showed no significant differences across all eight trials (Figure 2). The correlation coefficient and RMS differences in the measured maximal AT strain between the two methods were on average r = 0.88 (range: 0.82–0.94; *p* < 0.01) and 0.59% (range: 0.35–0.96%) respectively.

### 3.3. Sensitivity of the Proposed Method

**Figure 3:**
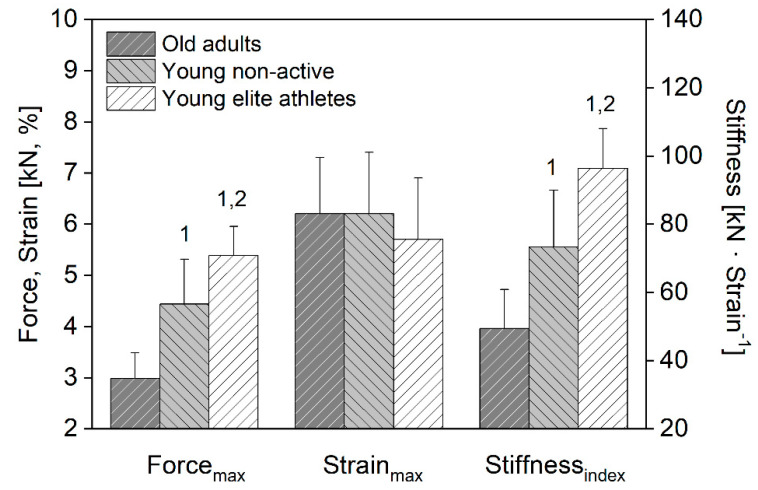
Means and SD of Achilles tendon maximal force, maximal strain, and stiffness index for the older adults (*n* = 8), younger non-active subjects (*n* = 11), and elite athletes (*n* = 8). ^1^ Statistically significant differences compared to the older subject group (*p* < 0.05). ^2^ Statistically significant differences compared to the younger non-active subject group (*p* < 0.05).

Maximal AT force was significantly (*p* < 0.05) higher for the elite athletes compared to the young non-active subjects and older adults (Figure 3). Further, the young non-active subjects demonstrated higher AT forces compared to the older adults (*p* < 0.05). There was no significant group-related effect on maximal AT strain (*p* > 0.05). Consequently, normalized AT stiffness index was significantly (*p* < 0.05) higher for the elite athletes (96.3 ± 11.7 kN · strain^−1^) compared to both other groups (Figure 3); and higher for the young non-active subjects (73.9 ± 18.3 kN · strain^−1^) compared to the older adults (49.4 ± 11.5 kN · strain^−1^).

## 4. Discussion

Ultrasonography has become a highly popular modality to assess the mechanical properties of human AT in vivo. However, since tendon elongation is usually estimated by manually digitizing tissue landmarks frame-by-frame using US, it is time consuming and not applicable where an immediate analysis is required (such as clinical and sports settings). In the current study, a time-efficient assessment of AT force, elongation, strain, and stiffness was developed. The results of three experiments suggest that the method is suitable for assessing human AT mechanical properties in vivo. However, to assure the reliability of the data, three to five trials should be analyzed in each test session.

The ICC analysis used in the current study for trial comparisons is considered as an appropriate statistical procedure to determine the reliability between measurements [24]. Moreover, the RMS and CV are proposed as valid methods to describe the deviation of a parameter in absolute and relative terms [24,25]. With respect to the maximal AT force, the overall ICC was close to excellent (r = 0.88) and the RMS and CV were low (421 N; 5.8%, respectively) confirming good reliability for this parameter. Although slightly lower, AT maximal strain and the stiffness index demonstrated an appropriate degree of reliability (see Table 2, Table 3 and Table 4). Moreover, it was found that the degree of within-day reliability for all parameters was higher than for between-day reliability. A reasonable explanation for this could be the re-application of the ultrasound probe and the LEDs on the following day. However, by analyzing two to three contraction trials at each session, reliable AT characteristics could be derived. Only for maximal AT elongation is a larger number of trials (about five) required to derive representative data.

Kongsgaard and colleagues [9] determined the between-day reliability for AT stiffness and reported a slightly higher correlation coefficient (r = 0.84). However, for their between-day comparison, they used the mean values of two trials per day with the highest AT force, thereby increasing the degree of reliability. When averaging our data over two trials (trials with the highest AT force), the strength of relationship in AT stiffness index between-day was similar to the data reported by Kongsgaard and colleagues [9] (r = 0.88, *p* < 0.01). In our study, the maximal AT elongation was assessed by digitizing one ultrasound frame at rest and one at maximal AT force, and the ratio of AT force to strain was used to assess the tendon stiffness. We compared this method with manual tracking of ultrasound images frame by frame and the absolute differences in tendon tracking was low (range in AT strain: 0.35 to 0.96%) and strength of relation between methods was high (0.82 ≤ r ≤ 0.94). Although it should be emphasized that this is an indirect validation of the method, the above data indicate an accurate means of assessing maximal AT strain during a plantarflexion contraction. Moreover, it seems that the current method is favorable to the usual methods described in the literature and has the potential to reliably and accurately assess AT mechanical properties in a time-efficient manner.

In addition, we were able to detect subject-group-related differences in both maximal AT force and stiffness index, with the elite athletes showing the highest, while the older adults the lowest values. The results fit to the existing literature and confirm the notion that habitual exercise with high AT strain magnitude increases tendon stiffness and muscle strength [2], and that the aging process is associated with a degeneration in muscle-tendon unit capacities [5,6]. The absolute differences in the normalized AT stiffness index between subject groups was on average 23 kN · strain^−1^, which is more than twice as high as the absolute error within-day (8.7 kN · strain^−1^) or between-day (10.6 kN · strain^−1^). This provides evidence that the current method is an alternative effective method and may be used for monitoring changes in AT mechanical properties resulting from altered mechanical loading and aging.

The calculation of AT stiffness using two frames (the ratio of AT force-to-strain) is the most obvious limitation of this work, discarding potential non-linearity of the force–strain relationship. Nevertheless, the force–length relationship of the Achilles tendon at the current ankle joint configuration during a ramp plantarflexion contraction is usually rather linear (no clear toe region, as the collagen fascicles are not in a slack position) [26,27]. Furthermore, one might argue for the use of an automatic tracking method, which, however, is still not time-efficient in comparison to the proposed two time point digitization and often requires further data inspection and correction [15]. The current method requires no high frequency time-series data for assessing tendon elongation as well as joint kinetics and kinematics, which is in favor for the use in applied settings where immediate reports are needed. Accordingly, since the proposed method was revealed to be reliable and was indirectly proven valid against conventional frame-by-frame tracking, in addition to being able to identify differences between groups, we suggest that the method can be used as a valid measure of AT mechanical properties.

## 5. Conclusions

In conclusion, our results demonstrate that the proposed method compared well with conventional frame by frame tracking and we were able to determine differences in AT mechanical properties between older adults, young athletes, and non-active subjects in a time-efficient manner. Moreover, the findings show appropriate within- and between-day reliability. Thus, the method proves to be an alternative effective technique to analyze AT mechanical properties in a time-efficient manner and can provide a helpful and practical approach, especially for clinical, sport, and scientific settings where immediate results are required. However, to assure an appropriate reliability, at least three contraction trials must be analyzed at each session to determine representative characteristics of human AT mechanical properties in vivo.

## Figures and Tables

**Table 1:** Means and (SD) for the analyzed parameters describing the mechanical properties of the Achilles tendon for the eight trials analyzed on day one (Trial 1–Trial 4), day two (Trial 5–Trial 8), and overall (Trial 1–Trial 8).

	Day 1				Day 2				Overall
	Trial 1	Trial 2	Trial 3	Trial 4	Trial 5	Trial 6	Trial 7	Trial 8	
Tendon force_max_ (N)	4324	4343	4369	4367	4588	4503	4575	4416	4436
	(1029)	(870)	(856)	(818)	(1005)	(935)	(922)	(987)	(898)
Tendon elongation_max_ (mm)	13.1	13.3	13.0	12.9	12.2	12.7	12.5	12.0	12.8
	(2.5)	(3.1)	(3.0)	(3.0)	(2.6)	(2.2)	(2.4)	(1.7)	(2.6)
Tendon strain_max_ (%)	6.3	6.4	6.3	6.3	5.9	6.2	6.0	5.9	6.2
	(1.3)	(1.5)	(1.5)	(1.5)	(1.4)	(1.1)	(1.2)	(0.9)	(1.3)
Tendon stiffness_index_ (kN/strain)	70.2	69.8	72.6	73.5	78.9	73.5	76.9	75.6	73.9
	(19.6)	(16.8)	(23.8)	(24.9)	(18.1)	(12.7)	(16.0)	(14.4)	(18.3)

**Table 2:** Coefficient of variation within day one, day two, and overall, for the analyzed parameters describing the mechanical properties of the Achilles tendon.

	Day 1	Day 2	Overall
Tendon force_max_ (%)	4.5	3.4	5.8
Tendon elongation_max_ (%)	6.7	5.8	10.3
Tendon strain_max_ (%)	6.8	5.7	9.7
Tendon stiffness_index_ (%)	7.1	6.4	10.1

**Table 3:** Root mean square differences within-day, between-day, and overall, for the analyzed parameters describing Achilles tendon mechanical properties.

	Day 1	Day 2	Between Days	Overall
Tendon force_max_ (N)	352	336	534	421
Tendon elongation_max_ (mm)	1.5	1.3	2.3	1.8
Tendon strain_max_ (%)	0.7	0.6	1.0	0.8
Tendon stiffness_index_ (kN/strain)	8.9	8.5	10.6	9.6

**Table 4:** Intraclass correlation coefficient values within-day, between-day, and overall, as well as the least number of trials (K) required within-day (measurement session) to provide reliable values for the analyzed parameters describing the mechanical properties of the Achilles tendon.

	Day 1	Day 2	Between Days	Overall	K
Tendon force_max_	0.92	0.94	0.90	0.88	1–2
Tendon elongation_max_	0.91	0.83	0.56	0.67	4–5
Tendon strain_max_	0.91	0.83	0.72	0.76	2–3
Tendon stiffness_index_	0.88	0.84	0.75	0.77	2–3

## Data Availability

The datasets used and/or analyzed during the current study are available from the corresponding author on reasonable request.
